# How Do Children and Adolescents with ASD Look at Animals? A Scoping Review

**DOI:** 10.3390/children11020211

**Published:** 2024-02-06

**Authors:** Manon Toutain, Nicolas Dollion, Laurence Henry, Marine Grandgeorge

**Affiliations:** 1 CNRS, EthoS (Éthologie Animale et Humaine)—UMR 6552, University Rennes, Normandie University, F-35000 Rennes, France; laurence.henry@univ-rennes.fr (L.H.); marine.grandgeorge@univ-rennes.fr (M.G.); 2 Laboratoire C2S (Cognition Santé Société)—EA6291, Université Reims Champagne-Ardenne, F-51100 Reims, France; nicolas.dollion@univ-reims.fr

**Keywords:** autism spectrum disorders, visual attention, youths, animal, human-animal interaction

## Abstract

Autism spectrum disorder (ASD) is characterized by interaction and communication differences, entailing visual attention skill specificities. Interactions with animals, such as in animal-assisted interventions or with service dogs, have been shown to be beneficial for individuals with ASD. While interacting with humans poses challenges for them, engaging with animals appears to be different. One hypothesis suggests that differences between individuals with ASD’s visual attention to humans and to animals may contribute to these interaction differences. We propose a scoping review of the research on the visual attention to animals of youths with ASD. The objective is to review the methodologies and tools used to explore such questions, to summarize the main results, to explore which factors may contribute to the differences reported in the studies, and to deduce how youth with ASD observe animals. Utilizing strict inclusion criteria, we examined databases between 1942 and 2023, identifying 21 studies in international peer-reviewed journals. Three main themes were identified: attentional engagement and detection, visual exploration, and behavior. Collectively, our findings suggest that the visual attention of youths with ASD towards animals appears comparable to that of neurotypical peers, at least in 2D pictures (i.e., eye gaze patterns). Future studies should explore whether these results extend to real-life interactions.

## 1. Introduction

### 1.1. Definitions

Autism spectrum disorder (ASD) is a neurodevelopmental disorder. It appears early in childhood and has significant impacts on individuals’ development throughout their lives. According to international classifications [1,2], ASD is characterized by two main areas of alteration: (1) deficits communication skills—both verbal (e.g., echolalia, impaired verbal fluency, delayed language development) and non-verbal (e.g., poor understanding and use of gestures, facial expressions, and turn-taking)—including deficits in language and joint attention, as well as little to no eye contact, all leading to restricted social interactions, interests, and bonding; (2) restricted and repetitive behaviors and interests, including verbal and motor stereotypies, generally accentuated by strong emotions [1]. Nowadays, understanding of ASD has evolved to embrace the concept of neurodiversity, emphasizing the presence of different forms of functioning rather than considering ASD a list of deficits. Individuals with ASD can also exhibit sensory specificities, which consist of hyper- or hypo-reactivity to sensory stimuli. For example, one individual with ASD can display various symptoms of visual hypo-sensoriality (e.g., being attracted visually by lights, looking intensely at brightly colored objects), while another may display hyper-sensoriality (e.g., focusing on tiny pieces of dust, mainly looking downwards, covers/shuts his/her eyes in the presence of bright lights) [3]. These sensory specificities have recently been included in the definition of ASD in the latest version of the DSM (5th edition; [1]). Since perceptual alterations can lead to a different sorting of the information flow (e.g., auditory, visual, tactile), this would lead to a different perception of the world.

### 1.2. Characteristics of Vision in Individuals with ASD

Vision is one of the most developed of the human senses. Our eyes provide us with up to 80% of our sensory perceptions and vision, thus having a major influence on how we integrate information from our surroundings and acquire knowledge about our environment [4]. However, as with other senses, processing visual information requires sorting. Indeed, throughout our daily lives, our vision is constantly solicited by a continuous flow of information from our environment. However, not all of this information is relevant. Part of it has to be selected and processed, while other elements have to be inhibited in order to reduce the amount and complexity of processed information and avoid informational saturation [5]. Such a selection process is notably enabled by attentional mechanisms. Our visual attention thus determines what we notice, which in turn determines how we may act according to the currently perceived situation [5,6]. From birth, visual perception plays a key role in the development of social interactions, as babies’ relationships with their surroundings are visual before being verbal [7]. Visual perception and attention to others are crucial to the quality of interactions we establish with our social environment and our peers [8]. Therefore, if the processes engaged in perception, sorting, or encoding of visual information are altered, this could entail developmental consequences. These visual information extraction alterations are often present in ASD individuals and have numerous consequences that can concern behavioral development (e.g., adaptability, acquisition of social norms), cognitive development (e.g., language, recognition, imitation, praxis), emotional development (e.g., empathy, recognition of emotions), or school achievements (e.g., learning to read and write) [7,9]. Interaction difficulties may be caused by the inability to visually detect, analyze, pay attention to, or understand the current environment or situation due to poor visual acuity (i.e., perceptual disorders of peripheral origin) or cerebral integration difficulties (i.e., alterations of lower to higher order functions—visual cognition) [7,9].

Concerning vision specificities of ASD individuals, the majority of authors agree that their visual acuity is not superior to that of typically developing (TD) individuals (children with Asperger Syndrome (AS) or with high-functioning ASD (HFA) [10], young adolescents with ASD, AS, or Pervasive Developmental Disorder Not Otherwise Specified (PDD-NOS) [11], adolescents and young adults with ASD [12]; and young adults with AS [13]). Authors studying color preferences reported that, just as TD children, boys with ASD prefer red and blue, and that both groups shun the color pink [14]. However, preference scores differentiated ASD from TD, with a lower preference for yellow among ASD individuals who tended to prefer green and brown. The authors hypothesized that some colors could cause sensory overload for children with ASD with heightened sensitivity. Another study on color vision showed that 30% of their sample of children and adolescents with ASD had difficulties discriminating colors [15]. Franklin et al. [16] reported that in visual search, memory, and target detection tasks, children with HFA detected color differences (between two types of red, yellow, and green) less accurately than TD children. However, their categorical color perception appears to be intact. Literature also reports that children with ASD present a bias for processing local features [17] compared to TD children who favor global processing (i.e., they extract information gradually and thus identify first the overall characteristics of a stimulus and then its finer details) [18]. Local processing may be advantageous in specific tasks (e.g., Figure Disembedding and Block Design Tasks [19]) but may also be a source of difficulties in others, such as facial expression processing, which requires holistic processing. Studies conducted on exogenous and automatic attentional orientation abilities of young children and adults with ASD did not reveal any significant differences between participants with ASD and TD subjects [20]. However, a more recent study showed that adults with HFA can present an alteration of attentional distribution [21]. Here, findings suggested an inability to process all of a visual scene simultaneously (i.e., to distribute their attention over a large visual field all at once). Additionally, several authors argued that the visual perception of children with ASD could be related to anomalies in brain areas dedicated to visual processing such as the magnocellular pathway involved in relaying information corresponding to the globality of a visual scene [7,22]. In the long term, all these visual specificities can contribute to interaction disorders and be overshadowed by them, hence the need to identify these disorders as soon as possible. 

As a result, studying how ASD affects visual attention as well as the perception of the surrounding biotic and abiotic worlds presents a real interest. Historically, children’s visual attention has been assessed using various age-adapted tools (e.g., specific comprehensive tests such as barrage and computer tests, or via self or hetero questionnaires), notably during clinical investigations. More recently, eye-tracking technologies (i.e., tracking and recording of eye movements via real-time calculation of gaze position) have revolutionized the measurement of visual attention by providing direct access to and recording of it. Nowadays, this technology is used in various fields (e.g., commercial, medical) and allows non-invasive, high-precision study of participants’ eye movements while they perform various activities (e.g., reading, searching for a visual stimulus) [23]. One of its greatest strengths is that it allows for the recording of data on children of all ages, even non-verbal, with or without explicit instructions [24]. A large number of scientific publications have used this technology to study individuals with ASD [23], and many of them have used it to investigate their visual processing of human faces and their interactions with them.

While visual attention evolves throughout life and can vary according to factors, such as age and gender. It is well documented that men are three times more diagnosed with ASD than females, which therefore implies that our current understanding of the female ASD phenotype is limited. A recent study conducted in 2019 shows a sex difference, with women with ASD showing a greater preference for faces than men with ASD, particularly in socially lean scenes (i.e., without social interactions) [25]. Additionally, ASD women often use compensatory behaviors to mitigate their social challenges, which adds complexity to identifying the challenges they may face during social interactions [26,27]. Concerning visual attention development (i.e., age factor), a longitudinal study conducted in children shows that at 6 and 9 months, infants with or without later diagnosis of ASD have similar skills, whereas differences are observed later, at 12 months old [28]. Nevertheless, the evolution of visual attention in children with ASD remains poorly documented in the scientific literature.

### 1.3. Understanding Human Partners: Perception of Social Stimuli

These visual specificities present in ASD could interfere with the collection of information and thus interfere with higher levels of integration, affecting the understanding of and response to everyday situations. Good visual information extraction concerning social partners is a prerequisite to social attention and to higher social skills, notably those engaging in face processing (e.g., face recognition, facial emotion recognition, intention attribution, theory of mind), which are essential to producing adapted and efficient social interactions [29]. Research concerning social information has also demonstrated that the visual behaviors of individuals with ASD present various specificities. 

Different studies have revealed that people with ASD focus less on the eye area and appear to look more at the mouth area when exploring faces [30], and the presence of various differences (e.g., in eye contact) have been widely studied [31,32,33]. In her book, Grandin [34] collected testimonies of individuals with ASD describing how they felt during eye-to-eye contact with another individual: “[...] when somebody looked at him in the eye, his mind went blank and his thoughts stopped; it was like a twilight state”, or “forced eye contact would cause her brain to shut down”. Furthermore, children and adults with HFA need to be taught how to develop facial expertise. In a study using faces and greebles (i.e., a novel class of perceptually homogenous objects), Scherf et al. [35] showed that children with ASD’s recognition abilities were reduced for both greebles and faces, suggesting that individuals with ASD have a generalized deficit in visio-perceptual processing, in particular configural processing, that could interfere with the development of a visual processing expertise, such as face processing. Additionally, some visual and social attention specificities are even included in the ADOS diagnostic tool [36] as being commonly observed symptoms: less spontaneous pointing initiation, difficulties in joint attention, difficulties in interpreting observed gestures, unusual eye contact, and difficulties in following others’ gaze [37]. Numerous studies reported children and adolescents with ASD’s differences in identifying and interpreting human facial expressions (e.g., [38,39,40,41]). Hobson et al. [42] found that children with ASD had difficulty matching photographs of basic target facial expressions with the same expressions displayed by a different individual, either on full faces or parts of faces. 

When asked to look at static social scenes (e.g., a mother and daughter sharing a meal), children with ASD look less at the characters and more at the background [43,44]. Similarly, when watching video clips, adolescents and young adults with ASD focus less on people, faces, and eyes than on other areas that are not directly relevant to social processing (e.g., objects, body regions; [30]). For many years, these atypicalities in visual perception and attention have been at the core of several hypotheses trying to account for the communication differences inherent to ASD. Some authors believe that these differences are due to alterations in specific abilities, such as the “theory of mind” [45], “executive dysfunctions” [46], or a deficit in “central coherence” [17]. While according to others, alteration of social motivation could be at the core of ASD specificities [47], since it results in social behaviors indicative of “little or no social interest” [48], or even in an aversion of social stimuli [49], including human gaze [34]. It is supposed that reduced orientation towards social stimuli from early childhood would lead to atypic abilities to build, learn, and develop social skills from positive or negative feedback from social stimuli, since the latter have little or no rewarding value [47]. In line with this last theory, ASD’s facial expression recognition difficulties have been shown to be present and stable during development [50]. A recent study in fMRI even reports that, despite comparable face recognition scores, adolescents with HFA showed hypo-activation of brain areas specialized for face processing (i.e., the neural network of the fusiform face area) in response to unfamiliar human faces [51].

### 1.4. Understanding a Non-Human Partner

As previously mentioned, the communication atypicity of individuals with ASD can affect both their language and non-verbal communication (DSM-V; [1]). Additionally, individuals with ASD also face various challenges and difficulties affecting their ability to engage in and maintain social interactions. As defined by Hinde [52], interaction consists of a reciprocal exchange of behaviors, limited in time, between two or more individuals. During a social interaction, atypical eye contact is one of the most evocative symptoms of social differences in ASD. Early descriptions of the symptomatology of ASD already highlighted this aspect [53], and it is now included in the latest version of the DSM diagnostic criteria (DSM-V, [1]).

It seems legitimate to think that these communication and social interaction differences could be generalized and apply to other forms of interactions, meaning that they would also be present in interactions involving either another species, any anthropomorphic agent, or even a robot. However, studies suggest that it is not necessarily the case, particularly with non-human animals.

While individuals with ASD’s empathy skills towards other humans are atypic [54,55], the situation seems to be different with animals. Using an online survey, Miralles et al. [56] asked 202 people with ASD to designate on pairs of photographs (i.e., pictures of 52 different species, including Homo sapiens) the organism they felt they were better able to understand the feelings and/or emotions. Empathy scores for the ASD group and for the TD group were similar across species (i.e., as phylogenetic distance from humans increased, the estimated empathic ability decreased) [56], except for empathy scores for humans. These were significantly lower for the ASD group, placing them at the same empathy level as reptiles and amphibians. Thus, it appears that empathy defects are not generalized to all living beings but could be specific to humans. In addition, adolescents with ASD recognized human facial emotions better when faces were transformed into an anthropomorphic chimera (e.g., a human face with a gorilla or lion outline) [57]. Furthermore, while adolescents with HFA showed hypo-activation when processing areas of unfamiliar human faces compared to TD individuals, no significant difference was observed for animal faces [51]. In a recent study of children with ASD, Davidson et al. [58] reported that children with ASD recognized facial emotion on human faces less accurately than TD children, no significant difference was observed when emotion recognition concerned dog faces. However, this specificity of animal information processing by children with ASD may not extend to all forms of information processing. Indeed, for example, children with ASD (5 and 6 years old) seem to have more difficulties judging an animal’s age than TD children [59]. Qualitative studies show that people with ASD can identify with animals more easily [60]. This is also reflected in terms of visual attention, as children with ASD look longer at animal faces (i.e., cat, dog, monkey) than at robot or human faces (familiar or not) [61].

Concerning other animated forms, recent studies suggest that people with ASD may be better at interacting with anthropomorphic stimuli than with more realistic stimuli. People with ASD seemed to be particularly attracted to anthropomorphic agents [60]. Anthropomorphic stimuli can take various forms, ranging from objects such as robots and dolls to more abstract forms, such as cartoons or avatars. Contrary to TD children, children with ASD are less sensitive to strange character modifications of anthropomorphic faces (e.g., eye size, [62]). Silva et al. [63] found that, while children and adolescents with ASD avoided emotionally positive stimuli when they were real pictures, they were attracted by them when they were cartoon images. Using eye-tracking, Saitovitch et al. [64] studied the visual exploration of children with ASD of video clips and pictures with either cartoon characters (i.e., Peter Pan cartoon) or human actors (Peter Pan film). As expected, children with ASD looked less at human eyes compared to control children, but spent an equivalent amount of time looking at the eyes of cartoon characters. Brosman et al. [65] observed that, concerning emotion recognition of human versus cartoon faces, adolescents with ASD performed better with static cartoon faces than the TD group. Another recent study demonstrated that adults with ASD were better at emotion recognition of cartoon faces than non-ASD individuals (RME cartoon version, [66]). 

Similar trends appear to apply to more artificial life forms (i.e., agents with fewer anthropomorphic features). A systematic review on this topic shows that robots are more socially attractive than humans for individuals with ASD [67]. In contrast, Hernandez et al. [68] found no difference between the exploration of avatar faces and other types of faces (i.e., pictures of Caucasian males) by adults with ASD. Furthermore, the same authors showed that children with ASD spent significantly less time focusing on avatars than age-matched control subjects. To conclude, concerning these more artificial forms of stimuli, no consensus emerges concerning the attractiveness and comprehensibility of these agents for people with ASD.

### 1.5. Perception: Mechanisms Contributing to Differences between Humans and Non-Humans

Whereas visual attention to humans has been widely studied, visual attention to animals has rarely been addressed. Consequently, despite the considerable impact that animals can have on children’s lives [69,70], the ontogeny of children’s visual attention and perception of animals remains poorly studied. Numerous reports showed that interacting with an animal is different from interacting with conspecifics and that it may influence beneficially various facets of children with ASD’s development: physical (e.g., facilitating motor development [71]), social (e.g., increased social motivation to communicate and pro-social behavior [72,73]), emotional (e.g., comforting, regulating feelings, and stress [70,71]).

Furthermore, studies showed that animals are attractive visual stimuli for children with ASD, both in 2D (animal images) and in real life interactions (animals in the room) [74,75]. When exposed to pictures of humans, animals and objects, children with ASD looked more at animals [74], while in a free interaction situation with an unfamiliar human, an unfamiliar therapy dog, or unfamiliar objects (i.e., toys), they interacted more with the animal [75]. It therefore seems that animals represent a source of motivation to engage in interactions (visually and vocally). In line with these observations, several studies show that while individuals with ASD have particularities in interactions with humans, these particularities are less marked when interacting with animals [76,77,78,79]. Several hypotheses have been proposed to explain these differences between animals and humans, one of which is based on the atypical or dysfunctional visual information extraction of human stimuli as an explanatory pathway [30,80]. Indeed, the atypical visual exploration pattern of children with ASD of human faces (i.e., random or inefficient exploration [81]) could contribute to less efficient information intake and processing, possibly leading to the expression of inappropriate behaviors during interactions. On the other hand, this atypical visual exploration pattern does not seem to be present when the interaction partner is an animal. It would therefore lead to more efficient information extraction, the generation of a better representation, and a reduction or absence of difficulties in an interaction with an animal [82]. As exposed earlier, Cross et al. [83] demonstrated that the simple addition of an animal filter on human faces (e.g., the addition of a lion or gorilla outline on a human face) was sufficient to improve people with ASD’s recognition of emotions. 

Initial studies using eye-tracking supported this hypothesis. Indeed, children with ASD explored more of the eye area of animal faces but not of human faces [80,84]. A recent study by Dollion et al. [85] using eye-tracking showed that having a service dog improved children with ASD’s face scanning strategies in a facial expression recognition task of human avatar faces. Despite the key interest of the eye-tracking technique in furthering our understanding of people with ASD’s visual exploration strategies and their visual world, this technology remains underused in studies of individuals with ASD. 

Animals represent particular interaction partners for people with ASD. We could argue that visual attention specificities may explain this specific status. Improving knowledge on this last topic will not only contribute to furthering our understanding of what contributes to the benefits of animals for individuals with ASD, but also to improving our understanding of the specific visual attention specifities related to ASD. The aim of this review is to present an overview of scientific research conducted on children and adolescents (i.e., a crucial developmental period for the acquisition of social, cognitive, and emotional skills) with ASD’s visual attention to animals. A scoping review was favored here to identify, summarize and evaluate all existing scientific studies and provide information concerning the most commonly used methodologies and animal stimuli, and thus provide guidance for future research. The more specific aims of this scoping review were: (1) to review the methodologies and tools used to explore such questions; (2) to try to organize and summarize the main results; (3) to explore which factors may contribute to the differences in the observations reported in the studies; and, lastly, (4) to identify the main limits of the studies and the elements that future studies should take into consideration in order to better identify the specificities of visual attention towards animals versus humans in children and adolescents with ASD.

## 2. Materials and Methods

### 2.1. Search Strategy

A scoping review is a tool for reporting the state of knowledge based on a body of literature on a topic. It highlights a body of studies and scientific evidence available on that topic [86]. It can also provide information on methods and practices in a given field and on how research was conducted [86]. This review was performed following PRISMA-ScR guidelines [87].

### 2.2. Search Protocol

Studies were collected from three common databases associated with the fields of biology science, psychology, health, and education (i.e., PubMed, ERIC (Education Resources Information Center) and WOS (Web of science)) and through supplementary searches using Google Scholar and Connected Paper. This search for articles started on 12 December 2021 and ended on 28 February 2023. Concerning the range of publication years, the earliest is from 1942 (date of the first appearances of descriptions of autism by Kanner and Asperger [88]), and the latest was published on 28 February 2023.

Our search strategy followed the following PRISMA guidelines:

(a) research question set up and definition of inclusion and exclusion criteria. Exclusion of records that were not relevant to the visual attention of young people with ASD, based on titles and abstracts; exclusion based on the full text when that article did not include measures of visual attention to an animal or exposure to animal stimuli in their living/realistic form [developed by all the authors].

(b) selection of databases [performed by MT, validated by other authors].

(c) selection of keywords. We searched for terms referring to four main categories: visual attention, target population (children/adolescents), autism spectrum disorder, and visual target (animals) (see details of the equation in Appendix A) [performed by MT and double-checked by all authors].

(d) search equation with keywords in databases (n = 336 references found) [performed by MT and double-checked by all authors].

(e) removal of duplicates on Zotero (n = 248) [performed by MT].

(f) application of inclusion and exclusion criteria, based firstly on the title, then on the full text (n = 18) [performed by MT and double-checked by all authors].

(g) final selection and additional search on Google Scholar, and Connected Paper websites to overcome a selection bias caused by keyword search (n = 3 more). Indeed, some of the selected publications based on keyword searches in this review may have had primary or secondary objectives that were different from visual attention to animals by children and adolescents with ASD. Selection bias could potentially occur due to the keywords selected and inserted in the search equation [performed by MT and double-checked by all authors].

**Figure 1:**
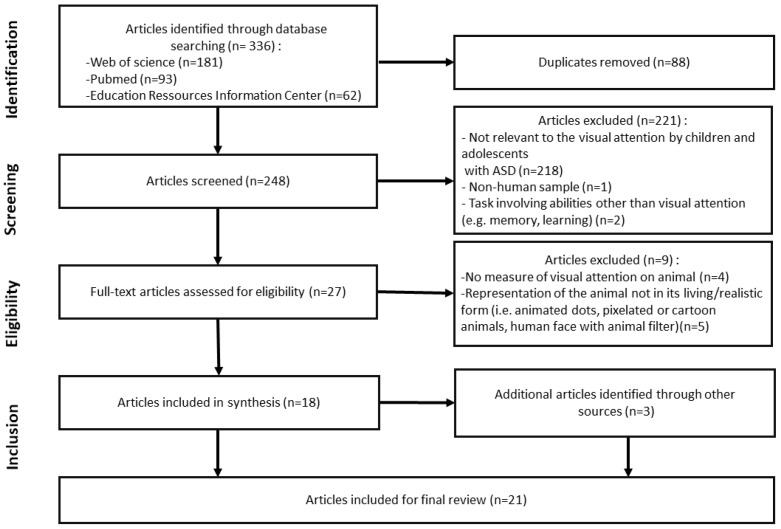
Flow chart of the study selection process.

Finally (h), on the basis of the final article selection (n = 21), the main results were extracted (see PRISMA flow chart on Figure 1) [performed by MT and double-checked by all authors].

No program was used to control and monitor the selection process.

### 2.3. Eligibility Criteria

The following inclusion criteria were applied: a study must (a) include measurement(s) of visual attention of youth people with ASD to animal(s) with the term “youth people” referring to all children and adolescents between the ages of 0 and 18 years-old; (b) use either real animal(s) or representations of them in a realistic form (i.e., use real photographs and not cartoons or dots representing biological motion); (c) be in English and peer-reviewed; and (d) report quantitative and qualitative results with the use of statistics. All selected studies were published in peer-reviewed journals. 

### 2.4. Charting Data

Only papers that studied the visual attention of children and/or adolescents with ASD to animals were considered. The search terms, inclusion criteria, and exclusion criteria were defined by consensus between the authors, after which some keywords were run through the HeTOP tool (https://www.hetop.eu/hetop/, Last accessed on 3 February 2024) (Health Terminology/Ontology Portal), which includes the main health terminologies and ontologies, in order to have a translation of the precise terms and synonyms. 

Information was extracted from each included study after final selection in order to achieve the following objectives of this review: (1) to review the methodologies and tools used to explore such questions; (2) to try to organize and summarize the main results; (3) to explore which factors may contribute to the differences in the observations reported in the studies; and (4) to identify the main limitations of the studies and the elements that future studies should take into consideration in order to better identify the specificities of visual attention towards animals versus humans in children and adolescents with ASD. For this purpose, information from the selected studies were divided into six distinct categories: publication characteristics (year, publication title, article title), participants’ characteristics (sample size, type of diagnostic, diagnostic measurement, age, gender, assessment of hypo- or hyper-sensitivity, pet ownership), methodological aspects (visual acuity check, children’s medication intake check, type of attention measurement, methodological details, duration of attention measurement, experimental setting, place of experimentation), control group (presence of control group, characteristics (diagnostic, age, gender)), stimuli characteristics (species/breed of animals, type of animal, presentation material, animal either known or not to the child), results and identified limits (main outcomes about visual attention, main limits mentioned). 

## 3. Results

### 3.1. Study Selection

The initial literature search yielded 336 articles, of which 248 remained after removing duplications. The final sample included 21 studies (Table 1). The flow diagram of the study selection process is presented in Figure 1. Due to the heterogeneity in the participants’ characteristics, study aims, methodology, and measurements in the selected articles, this review can only present a descriptive and qualitative synthesis rather than a meta-analysis.

### 3.2. Characteristics of Publications

Although many articles have been published in recent years on both visual attention and the benefits of animals for people with ASD, only a few focused on the visual attention to animals of children and adolescents with ASD (Table 1). Thus, only 21 scientific papers emerged from this literature search (8.4% of the initial pool).

The articles addressing this specific topic were published between 2010 and 2022, with at least one publication each year since 2010. The majority of these publications (12 papers out of 21 [82,83,84,97,98,99,100,101,102,103,104,105]) were published during the last 5 years, with an acceleration in publication speed during the last years (i.e., from 2 papers per year in 2018 [97,98], to 4 papers in 2019 [83,99,100,101], and 3 papers in 2020 [84,102,103]); then down to 2 papers in 2021, and finally one in 2022 (deceleration probably due to the COVID-19 health crisis in 2020 and 2021).

These 21 publications were published in 17 different scientific journals, with five journals represented twice. A diversity of publication scopes and publishing houses interested in this topic were also present: zoology and veterinary science (1), psychological discipline (1), health (1), psychiatry (2), developmental psychology and cognitive neuroscience (4), specific to human-animal interactions (2), specific to autism spectrum disorders (3), or interdisciplinary mega-journals (3).

The smallest sample size was five participants [83,101] and the maximum was 98 [97] (Table 2). The mean number of participants per study was 24.1 ± 22.2.

Most of these studies established diagnosis based on diagnostic tools (n = 13), in which case only two recurring diagnostic tools were used: ADOS (n = 9) and ADI-R (n = 11), which are tools internationally recommended for diagnosis (e.g., HAS, 2018 [106]). Most of the studies used additional tools for ASD assessment (i.e., 71.4%; e.g., CARS, SCQ, SSRS, SBRI, ICD-10, SRS-2, AQ). The diagnoses were confirmed in all studies except Avila-Alvarez et al.’s [103], in which part of the study population (n = 4) had a probable ASD, and in the particular case of a study using a cohort of high-risk children (diagnosis established after experiment) [97]. We noted that 9 of the 21 publications mentioned the participants’ IQ. Moreover, 15 publications used scales to characterize the severity of ASD. Participants’ ASD severity ranged from ‘mild’ to more ‘severe’.

Ages of participants ranged from 6 months to 14 years old, with 4 studies on pre-school children (6 mo–3 yo), 1 with children around 5 years old, 14 with children 6–12 yo, 1 with adolescents 13–16 yo, and 2 with both children and adolescents 7–14 yo.

Boys outnumbered girls, which is consistent with the unbalanced sex ratio in the ASD diagnostic (DSM-V; [1]) average of 82.2 ± 0.13% of boys, with a range from 50 to 100%. Three studies included only boys in their sample [91,98,107] without explicitly specifying any rationale for it. Only three publications mentioned checking for the presence of sensory processing disorders. Two of these three publications used this factor as an exclusion criterion, and only one specified the exact number of participants with sensory processing disorders.

Only seven publications mentioned the presence or absence of pets in the participants’ family households. One of them mentioned that none of the participants had a pet. Most of the participants in the other six publications had pet(s) (mean, 62.7 ± 0.35%). Only three studies specified exhaustively the species of the pet [99,102,103]: dogs, cats, rabbits, and guinea pigs.

### 3.3. Methodological Aspects

Before experimentation, (1) only seven studies reported taking participants’ visual acuity into consideration and/or checking it and (2) only two studies mentioned the presence of medication taken by subjects (Table 3). Strathearn et al. [98] specified the general type of medication (i.e., 69% of the children and adolescents with ASD were taking psychotropic medications), and McParland et al. [90] specified that medication intake was an exclusion criterion in their study.

A diversity in the methodology applied to measure visual attention is highlighted here. The more recurring methods were ethological methods (n = 8) and eye-tracking methods [108] (n = 8). Other methods are seldom used: questionnaires (n = 3), tests/tasks (i.e., films or images on screen and touch-screen response; n = 4).

**Table 3:** Summary of methodological characteristics.

Authors	Visual Acuity Check	Type of Attention Measurement	Details of the Type of Measure of Attention to Animals	Duration of Attention Measurements (min; s)	Scenario Conditions and Place of Evaluation
New et al. [89]	NA	Responses to a computerized test: mouse click responses	Performance in change detection (speed and accuracy): index of automatic attentional prioritization for each type of object that changes between two pictures. The changing target in each scene was either an animate object (person or animal) or an inanimate object (plant or artifact) either with reversed orientation or that repeatedly disappeared and reappeared.	14 images containing animals’ pictures out of a total of 56 images; 0.5 s for the first apparition of the image and until 20 s for the detection	Laboratory (room)
McPartland et al. [90]	Presence of eyeglasses as an exclusion criterion (n = 1)	Eye-tracking	Proportion of total fixation duration spent on each AOI (i.e., upper and lower areas of stimuli: mouth and eye position). AOI sizes and positions were equivalent across stimulus categories. 240 Hz.	10 animal stimuli out of a total of 50 stimuli, 8 s of apparition	Laboratory (room)
Grandgeorge et al. [91]	NA	Ethological methods	Looking at and glancing at one of the target items (i.e., parent, observer, animal, or environment). Binary variables (presence/absence).	15 min of exposure to an animal	Naturalist (at home)
O’Haire et al. [92]	NA	Direct observation (coding system = The Observation of Human-Animal Interaction for Research)	Gazing at (either a human animal or toy). Binary variables (presence/absence) during each 10-s interval over minutes pre-selected for coding.	Three sessions of 10 min exposure to an animal. Measurement of the first 10 min, including 3 min selected for coding	Naturalist (outside the school classroom)
Guillon et al. [93]	Yes	Eye-tracking	Number of first fixations on the left or right visual hemifield on stimuli (i.e., faces, objects). Number of non-lateralized fixations. A laterality index of the first fixation was used. 60 Hz.	12 animal stimuli out of 28; 3.5 s of apparition	Laboratory (room)
Grandgeorge et al. [94]	NA	Ethological methods	Gaze (frequency in %) directed at: guinea pig, human observer, parent, objects—either unfamiliar or familiar—or self-centered.	15 min of exposure to an animal. One exposure was stopped after 12 min	Naturalist (at home)
Muszkat et al. [95]	NA	Eye-tracking	Total number of fixations and total fixation time on two AOIs of equal size on facial stimuli (i.e., eye and mouth of either a dog or a human face). 300 Hz.	NA number of animal pictures, 5 s of apparition	Laboratory (room)
Grandgeorge et al. [80]	Vision acuity was an exclusion criterion: it had to be normal or corrected-to-normal vision (checked using the Monoyer’s scale of visual acuity).	Eye-tracking	Mean duration of fixation on three AOIs on each face (i.e., eyes, mouth, and ears for animals). The data were adjusted for variance in AOI size between stimuli. 60 Hz.	Six pictures of each animal, 5 s of apparition	Laboratory (room)
Grandgeorge et al. [96]	NA	Ethological methods	Gazes and glances (<1 s) occurrence and duration (s) based on eye/head orientation toward different targets (i.e., service dog, animal trainer, service dog and animal trainer dyad, environment including objects). Occurrences of joint attention were also collected.	S1: 30 min S2: 20 min of exposure to animals	Naturalist/Semi-standardization (room)
Doherty et al. [97]	NA	Response on touch screen	Accuracy and total time to complete the task. For each trial, participants had to touch the same type of target (animals) and avoid distractors (inanimate objects). three additional measures were produced: (1) Q score (2) best R (3) intersections rate (Score Q: “a measure of search efficiency combining speed and accuracy”, Best R: “a measure of horizontal or vertical spatial organization”; Intersections rate: “number of times the search path crosses over itself, divided by the amount of cancellations that are not immediate revisits” [109].)	No time limit for a trial; the trial ended when children touched 18 of the 20 targets (i.e., cats or animals or dogs) or emitted 40 responses, including some of the 70 distractors	Laboratory
Strathearn et al. [98]	Visual impairment was an exclusion criterion	Eye-tracking	Total duration and number of fixations on four AOIs on four pictures with different levels of organization (i.e., least systemized, less systemized, more systemized, most systemized). 60 Hz.	NA animal stimuli, 12 s of apparition	Laboratory
Germone et al. [99]	NA	Direct observation (coding system = The Observation of Human-Animal Interaction for Research)	Timed interval behavior coding system designed to quantify social communication and interactions with animals and control objects, including animal-directed gaze.	10 min with an animal, including 3 min selected for coding	Laboratory (open classroom)
Uccheddu et al. [83]	NA	Parent questionnaire	Including a question about the child’s attention towards dogs: “Was the child able to pay more attention to dogs in daily routine?” Answers: Yes/No.	30 min of exposure to an animal	Laboratory (room)
Gale et al. [100]	NA	Response on touch screen	Frequency of touch on each image (i.e., indicate preference) for each session. Pixelated movies of non-social stimuli (six different movies of abstract geometric moving patterns) and social stimuli (six different movies of dogs’ faces).	Six animal stimuli; if an animal was selected, it appeared for 1 min 30	Laboratory (child’s home, nursery, or clinic setting)
Yamashiro et al. [101]	NA	Eye-tracking	Total length of fixations in each oval AOI corresponding to a face or non-face stimuli (i.e., two human faces, two monkey faces, and their corresponding non-face stimuli presented simultaneously). 60–120 Hz.	Two pictures of an animal. Each trial lasted until the infant accumulated 10 s of looking time at either display or for a maximum of 20 s	Laboratory (room)
Grandgeorge et al. [102]	NA	Parent questionnaires and ethological methods.	Parent-based short questionnaire: frequency of visual interaction between their child and their pet (never, rarely, often). In addition, observation at home: glance, mutual gaze, gaze towards the animal.	60 min session of observation	Naturalist (at home)
Avila-Alvarez et al. [103]	NA	Questionnaire for health professionals	Brief questionnaire (Animal-assisted Therapy Flow Sheet). A scale was applied through direct observation of three sessions by an experienced health professional. It included frequency of «Looked at dog» (never, once, two or three times, and several times).	One session with an animal for around 20 min. Measurements were performed on one of the three sessions the child attended	Naturalist/Semi-standardized approach (room)
Valiyamattam et al. [84]	Yes	Eye-tracking	Gaze fixation durations on six AOIs on each human and animal face (i.e., face, left eye, right eye, eye region, mouth, and screen). Face AOIs were defined using the following landmarks: human hairline, ears, and nose tips. 120 Hz.	20 pictures of animals, 5 s of apparition	Laboratory (room)
Dollion et al. [82]	Yes	Eye-tracking and Ethological methods	Number and duration of fixations in six AOIs on the visual scene: the whole service dog, the service dog’s head, the evaluator’s head, the parent’s head, the board games, the service dog’s accessories, and the rest of the visual scene. Each AOI was defined to encompass the entire element of interest (+additional space around the target). Additionally, based on observation, occurrences and durations of gazes directed towards: parent, service dog, board games, service, dog’s accessories, other directions. Eye-tracking glasses at 60 Hz.	S1: 20 min of exposure and measurement S2: eye-tracking measurement: 4 min; observation: 20 min of exposure and measurement	Laboratory (room)
Scheerer et al. [104]	Yes	S1: Response on the keyboard S2: Response on the keyboard and eye-tracking	S1: accuracy and response time in an attentional capture paradigm (i.e., detect whether a target butterfly was present or absent among distractors (face, train, fruit, objects, etc.)). S2: total number of fixations and latency of first fixation to an AOI (i.e., butterfly, neutral stimuli). AOIs were defined as a 3 cm by 3 cm region around the stimuli. 1000 Hz.	S1 and S2: one trial: 0 to 2 pictures of animals among six stimuli. 120 trials total, with an apparition until the subject’s response or 5 s maximum	Laboratory (room)
Dollion et al. [105]	NA	Ethological methods	Gaze (frequency in %) directed at: service dog parent games and objects other.	20 min of exposure to an animal	Laboratory (room)

Legend: AOI = Area of Interest, NA = Unknown.

According to the methods used, publications provided details concerning visual attention measurements. When using ethological methods, researchers measured gaze (i.e., duration of more than one second [110], (n = 7) and glances (i.e., duration of less than 1 s [110], (n = 3). To do so, different sampling methods were applied: scan sampling, focal sampling, or presence/absence of behaviors. We noted that the authors of two publications developed a specific observation method: The Observation of Human-Animal Interaction for Research [92,99]. The questionnaires presented in some publications were created especially for the purpose of the study: the Animal-Assisted Therapy Flow Sheet (by a professional) measuring, for example, the frequency of looks at a dog during the experiment [103] and the short parent questionnaires measuring, for example, the frequency of gazes between pet and children [102] or children’s general visual attention to the dog in their daily lives [83].

Three of the four publications based on tests and tasks on computer used performance measures based on response time and accuracy [89,97,104]. New et al. [89] added an index of automatic attentional prioritization. Gale et al. [100] also used one screen and one response per screen touch, and did not assess performance but preference via the frequency of touch on the preferred stimulus.

Studies using eye-tracking (n = 9) presented classic data collected on Area of Interest (AOI): proportion of fixations (n = 1), total duration of fixations (n = 6), mean duration of fixations (n = 1), total number of fixations (n = 4), first fixation (n = 1), and latency of first fixation (n = 1). All these data were collected by defining AOIs on the stimuli in all papers, except one by Guillon et al. [93], who studied first fixations on the right or left hemifield via a laterality index calculation. The sampling of studies using eye-tracking ranged from 60 Hz to 1000 Hz. 

The number of AOIs used in the studies varied from 1 to 7, depending on the experimental design. The stimuli used by all of the eye-tracking publications, except for three publications, used photographs of faces. Dollion et al. [82] used eye-tracking glasses in a real-life interaction context (scenes including faces but not exclusively). Strathearn et al. [98] used photographs of more or less systematized stimuli for classification (one AOI per photograph). The authors define systematization as the level of organization of the image (e.g., a high level of systematization means that the elements in the scene are highly organized). Lastly, Scheerer et al. [104] presented stimuli consisting of arrays of six items (1 AOI per item) on a screen. The number of AOIs varied according to the level of accuracy on the faces; Yamashiro et al. [101] used only one AOI encompassing the entire face. This publication is the only one to offer children with ASD two faces simultaneously: either two faces or one face and a non-face stimulus. This strategy allows the measurement of a preference between the two presented faces. The other publications, including faces, defined different AOIs within the face: at least two AOIs (i.e., eye, mouth [90,95], three AOIs (eye, mouth, ears [80], or six AOIs (face, left eye, right eye, eye region, mouth, and screen [84]). When several stimuli were presented simultaneously in a real scene, as in Dollion et al. [82], one AOI was defined for each human face (i.e., parent, evaluator) and two AOIs for the dog (i.e., one AOI for the head and one AOI encompassing the whole dog). 

Six of the seven publications using AOIs mentioned how AOIs were delineated (i.e., equivalent size and position between stimuli of the same category or described only one of the AOIs). Only three publications provided more details by specifying the exact dimensions or their definition criteria or by mentioning at least one example (i.e., [80,82,104]; respectively). Two publications mentioned the shape of their AOIs (i.e., oval [101]; square [80]).

All experiments had different durations. The shortest duration of exposure was 0.5 s [89], and the longest was limited to 1 h [102]. The duration of exposure to animal stimuli varied greatly (0.5 s to 60 min). Durations varied mostly according to the type of stimuli used. When they were real animals, durations varied between 20 and 60 min. The authors of four of these publications proposed a 20-min exposure to a real animal [82,96,103,105], two proposed a 15-min exposure [91,94], two proposed a 10 min exposure, and one proposed an exposure for up to 60 min [102]. When the stimuli were pictures of animals, the durations were shorter. Some authors applied a fixed duration per picture: 3.5 s [93], 5 s [80,84,95,104] or 8 s [90]. One publication used videos of animals lasting 1 min 30 s [100]. Finally, the authors of the remaining publications applied non-fixed durations of stimulus presentation, i.e., between 0.5 and 20 s [89] and between 10 and 20 s [101]. Finally, the authors of one paper had no limited duration of exposure [97].

The number of animal stimuli presented during each experiment varied from one for real animal exposure (n = 10) to two animals per encounter. The numbers of different stimuli used for computer-based tasks varied from 1 to 20 different stimuli of animals (2 stimuli: [101,104]; 3 stimuli: [89]; 6 stimuli: [80,100]; 10 stimuli: [90]; 12 stimuli: [93]; 20 stimuli: [84]. A large majority of the studies (n = 8) presented one stimulus at a time. We noticed that three publications did not mention the number of animal stimuli presented to the subjects [95,97,98]. 

Other methodological characteristics, scenarios, and places of experimentation varied. Here, 15 publications were conducted under laboratory conditions and six under naturalistic conditions. Moreover, 11 of the 15 under laboratory conditions took place in a dedicated room. Two of the others took place in a classroom, three at the subject’s home, and one in a space that was familiar to the subject (i.e., home, nursery, or clinic setting); all places corresponded to more naturalistic settings, and most of them were known to the participants.

The majority of the publications included a control group (18 of 21). Thirteen studies observed a sample of neurotypical individuals. The control groups for the other studies consisted of other individuals with ASD (n = 3), individuals with ADHD and TD (n = 1), or individuals with a low risk of being diagnosed with ASD (n = 1). The average number of control participants was 26.3 ± 18.4, ranging from 4 to 66. Only four publications had the same sample sizes for the ASD group and the control group [83,90,95,98]. Ages of the control groups ranged from 6 months to 14.5 years old, and 15 of the 18 publications had a control group with less than one year of age difference with the ASD group (three have more than one year difference; e.g., [95,100,102]. Note that one publication included an ASD adult control group (i.e., students) in addition to the ASD child control group [89]. Chronological age was the most commonly used variable to describe control populations. Lastly, sex ratios were similar between ASD and control groups in only three studies [83,96,98]. Only nine of the publications mentioned an IQ measurement.

The species and breeds of the animal stimuli included in these studies varied greatly, i.e., 13 different species (Table 4). The most common stimuli were dogs, which were involved in 13 studies: seven in real life conditions (five studies specified breeds with Labrador, golden retriever, Border Collie, King Charles Spaniel Mix, Galician Shepherd Dog, Spanish Water Dog breed, Labernois, St-Pierre), one on videos, and five on pictures (three used black and white pictures). Only three studies detailed the dogs’ ages: around 2 years old on average [82,105], 5 years old on average (range between 2 and 8 years old [83], and 9 years old on average (7 to 13 years old [99]. Four studies used cats: one in real life conditions and three using pictures (one with black and white pictures). Three used guinea pigs (all in real-life conditions) and two used horses (both used pictures, with one using black and white pictures). All the other species were presented as pictures: monkeys (n = 3), common elands (n = 1), pigeons (n = 1), cows (n = 1), bears (n = 1), camels (n = 1), anseriforms (n = 1), butterflies, and fish (n = 1).

As previously stated, 10 of the 21 publications involved real interactions with an animal with only three different species used (i.e., seven with dogs, three with guinea pigs, and one with cats). Only one publication involved real interactions with two different species (dog and cat [102]. The 11 remaining publications presented animals in 2D (presentation on a computer screen), 10 of which used pictures and 1 used movies. Six of these eleven publications used animals’ faces only. 

Seventeen of the twenty-one publications used stimuli from animals that were unknown to the ASD subjects. Only one used an animal known to the subjects prior to the research (i.e., used their own pet [102]). Finally, three used an animal initially unknown to the subjects that then became known, notably because the experiment involved several encounters with the same individual (e.g., animal-assisted intervention sessions).

### 3.4. Main Outcomes Results about Visual Attention

Given the variability of visual attention measurements used in the selected publications, this review therefore focuses on a descriptive and qualitative synthesis of the main outcomes of the studies with animal stimuli. Therefore, the main observations and conclusions of the studies in each sub-theme have been classified as described below.

#### 3.4.1. Attentional Engagement and Detection

Three publications were interested in these visual attention components [89,97,104]. New et al. [89] showed that children with ASD were better at detecting changes in animal stimuli than in plant or object stimuli. The results of this study show that an attentional bias for the animal category was present. The cancellation task (i.e., ability to inhibit distractors in an array) used by Doherty et al. [97] showed that ASD is associated with a more disorganized and less successful visual categorical search, even when the stimuli were animals. Lastly, Scheerer et al. [104] showed that target detection was more accurate but slower when the butterfly target was absent compared to when it was present. When the butterfly target was absent, the distractors were fixated more often. Finally, children with ASD took longer to detect the butterfly and fixate it than TD children.

#### 3.4.2. Visual Exploration: Visual Preference for Humans versus Animals versus Others, i.e., What Do Children and Adolescents Look at?

Eight publications could be included, four in real life conditions [82,91,92,105] and four with animal pictures and videos [84,95,100,101].

In real life conditions, when children with ASD encountered an unknown guinea pig, most of their first gazes were at the animal rather than at the environment or other humans (66.7%; similar to TD children) [91]. In more standardized interactions, children with ASD looked at the toys more often than at the guinea pigs [92]. Children with ASD spent more time looking at the service dogs, and in particular at their faces, than at other elements of the visual scene [82]. Children with ASD who looked more at social/animate targets showed more engagement with the service dog. Conversely, when they looked more at non-social/inanimate targets, they showed less engagement. Lastly, the service dog was the preferred target of ASD children’s gaze during the observations, although the youngest children looked less at it than the older children [105]. This study also highlighted two profiles for interactions with a service dog: one group of children with ASD interacted with it distally and looked less at it, while the other group interacted more proximally and looked more at it. 

One study, using pictures and movies of animals in their experiments [95], showed that ASD and TD children looked more at dog faces than at human faces. A second study by Yamashiro et al. [101] showed that, whereas infants with ASD preferred human faces to non-faces, they did not prefer monkey faces to non-faces until they were 9 months old. Between 6 and 18 months old, the preference and total gaze durations at primate faces of children with ASD declined. Altogether, these findings suggest that a decline in attention to primate faces was present after the first year of life. In Gale et al.’s [100] study, when children with ASD were asked their preference by clicking on movies on a tablet depicting either non-social stimuli (geometric images) or dog faces, children with ASD preferred the non-social stimuli. Lastly, Valiyamattam et al. [84] reported that children with ASD paid greater visual attention to animal faces than to human faces.

#### 3.4.3. Visual Exploration: Visual Exploration of Animal Faces 

Five publications using an eye tracker could be included [80,84,90,93,95]. The first showed that ASD and TD children looked more at the upper half than at the lower half of primate faces [90]. When looking at human or canine faces, children with ASD presented no gaze bias towards the left hemifield of the face, unlike TD children [93]. The emotional valence of the displayed facial expression had no impact on this result [93]. Children with ASD looked more at the eyes than at the mouths of dog faces than at human faces, but the durations of eye fixation were lower [95]. No preference for the left visual hemifield was observed in TD children. Grandgeorge et al. [80] showed that TD children looked longer at the eye area than ASD children, whatever the picture presented. The only differences were that children with ASD looked more at the cat’s eyes, less at human faces, and more at humans’ mouths than at animals’ mouths than TD children. Finally, when looking at dog, cat, or horse faces, children with ASD paid more visual attention to the eye area than to the mouth and ear areas. Lastly, Valiyamattam et al. [84] revealed that children with ASD looked less at the face as well as to the left and right eye and the mouth of animals (dog, cat, horse, cow) and humans than TD children. Children with ASD showed greater visual attention to the eyes of animals than to human faces. When children with ASD looked at a human face, they looked more at the mouth and at the rest of the screen than they did at animal faces. Analyzing face orientation showed that children with ASD looked more at the right eye of faces facing them, whereas they looked more at the left eye of averted faces. In contrast, face orientation did not induce any differences between the ASD and TD groups concerning the mouth area.

#### 3.4.4. Visual Exploration: Visual Exploration in Relation to Species

Two publications were included [80,102]. Using an eye-tracker, Grandgeorge et al. [80] showed that children and adolescents with ASD looked more at the eyes than at other parts of the face, whatever the species considered (i.e., dog, cat, horse). However, when presented with dog and horse faces, they looked more at the eyes, then the mouth, then the ears, whereas the reverse was observed for cat faces. 

In real life interactions of ASD and TD children with their pets (dog or cat), Grandgeorge et al. [102] showed that TD children glanced more at cats and gazed more at dogs. In comparison, children with ASD showed greater attention to their cat, as they performed more gazes and glances at it than with their dog. Parents, answering an additional questionnaire, reported that both ASD and TD children had fewer visual interactions with their cats than with their dogs.

A final study, not directly manipulating species, can be cited in this aspect of image exploration because it used a preference paradigm involving different degrees of spatial organization of the same animal stimuli (i.e., anseriform). Strathearn et al. [98] showed that children with ASD had a higher fixation time on pictures with higher levels of systemization (i.e., more organized), a trend that was not observed in TD children. After receiving oxytocin, this preference was reduced for the most systematized images. We noted that no details were given for the anseriform images in particular. 

#### 3.4.5. Behavior

Eight publications focused on ASD children’s visual behaviors during a real interaction with an animal [82,83,91,92,94,96,99,102,103,105]. These studies resorted to: 1) direct observation using either classic ethological methods (n = 6) or the OHAIRE, a specific coding system based on direct observation (n = 2), or 2) questionnaires (n = 3, including two parental questionnaires and one health professional questionnaire). The species involved were dogs (n = 7, including three with service dogs, three with therapy dogs, and one with a pet dog), cats (n = 1 with a pet cat), and guinea pigs (n = 3).

Grandgeorge et al. [96] revealed that, when involved in a usual AAI session with a service dog (i.e., a dog trainer trying to involve a child in shared activities with a service dog, with attention focused on the child-service dog dyad), children with ASD only performed a few gazes and glances at both the dog trainer and the service dog. On the contrary, when a social rivalry situation was established (i.e., exclusion of the child with ASD from interactions—including visual ones—between the dog trainer and the service dog), children with ASD made more alternating glances between the service dog and the dog trainer when they were far from them, while at shorter distances, their glances were directed more at the “trainer-dog” dyad. Repetition of this procedure showed the maintenance of increased visual attention in children with ASD to the service dog-animal trainer dyad. During a social rivalry situation in the third session, children with ASD decreased their gazes at the service dog but increased them at the trainer-dog dyad. Their attention to the dog trainer increased, and the target was the service dog 85% of the time. During less controlled interactions with a service dog and using gaze time-budget, Dollion et al. [82] highlighted that children with ASD spent the majority of their time looking at the service dog (mean percentage of fixation duration: 44.4 ± 3.2%), and notably its head (31.4 ± 2.5%) compared to all other visual targets (e.g., the evaluator’s head or the parent’s head). 

By analyzing a similar situation Dollion et al. [105] identified two interaction profiles of children with ASD concerning their behavior during their first encounter with a service dog, notably based on their visual attention. One group of children interacted proximally with the service dog, with more gazes at it as well as at their parents (74.7 ± 7.6% vs. 11.4 ± 3.7%), and another group remained more distant from the service dog. An age effect was found: the older the children with ASD were, the less they looked at objects and toys, and the more they looked at the service dog. 

Measures of interactions with therapy dogs were either performed directly during AAI sessions or deduced from questionnaires. Uccheddu et al. [83] collected parents’ opinions concerning their child with ASD’s attention to therapy dogs from the first session of reading through a dog program. No significant difference was found concerning children’s attention to the therapy dog, depending on whether or not the children with ASD were exposed to the therapy dog during the reading session. Germone et al. [99] showed through direct observation that the quantity of gazes at a target varied in relation to the stimulus (toy versus dog). Children with ASD looked more at a toy than children with ASD with the therapy dog (mean counts: 16.1 ± 0.5 vs. 14.1 ± 0.6). Lastly, Avila-Alvarez et al. [103] showed that visual contacts of children with ASD with a therapy dog increased in frequency across AAI sessions (mean rank: first session vs. final session: 1.63 vs. 2.16). 

Grandgeorge et al. [102] described the visual interactions between ASD and TD children with their pets (dogs and cats). Comparisons between ASD child-dog and ASD child-cat dyads showed that their attention structures did not differ. Analysis of the structure of attention during interactions with cats showed that mutual gazes between children with ASD and their cats were rare; however, children with ASD performed more gazes and glances at their cats than with their dogs. When the stimulus was a guinea pig, it was the first target looked at during a first encounter with it by both children with and without ASD [91]. Almost three-quarters of the children with ASD looked at the guinea pig. Focusing on types of behaviors displayed with pets, Grandgeorge et al. [91] stressed that children with ASD looked more at the animal than they touched it (25.1 ± 12.9% vs. 21.5 ± 15.3%). The direction of a child’s gaze during an encounter showed that they looked more at the pet than at other elements in the visual scene (i.e., parent, observer, unfamiliar objects, familiar object, self) (the guinea pig accounted for 48.8 ± 17.2% of the scans). Lastly, O’Haire et al. [92] showed that during free interactions with a guinea pig, children with ASD looked more at the toy than at the animal. 

### 3.5. Limits

We focused here on the limitations the authors mentioned themselves in their studies. Four studies did not mention any limitations [89,91,96,101]. The most frequently mentioned limitation was the small sample size (n = 5; [80,82,83,95,104,107]. The second most mentioned limitation was the lack of information characterizing the sample (e.g., cognitive functioning, IQ [92]; manual and ocular dominance [93]; comorbidity [99]; severity of disorder [103]; presence of animals at home [105]). Six publications addressed limitations relative to the applied protocol. It concerned, for example, the availability of stimulus resources [90], the fact that results cannot be extrapolated [83,94], the fact that the same subjects participated in the three studies [100], stimulus attraction bias [82,105], effects of fatigability and stimulus order [97], duration [80,103] and the fact that the task was too simple for the participants [104]. Three publications mentioned the size of the AOIs [80,82,90] and the characteristics of the stimulus as limitations [80,99,100]. Some limitations related to participants’ characteristics were also addressed, e.g., no mental retardation [80], age of individuals [104], and reduced range of ASD severity [105]. Less often, limitations related to the tools used were addressed, such as questionnaires completed by the children’s parents [83,97] or the use of an earlier version of a tool [82]. Lastly, occasionally other limitations were found, such as a lack of a control group [103] and an unbalanced sex ratio (e.g., a higher number of boys [84]).

## 4. Discussion

Accurate and efficient perception of and adaptation to our social and non-social environment depend on our ability to extract and integrate information from it correctly, notably through visual attention. While numerous studies have demonstrated that both individuals with ASD’s social interactions and visual attention to social stimuli (i.e., human partners) are altered, recent studies revealed that this did not seem to be the case for pseudo-social stimuli (i.e., animals). Over the past 13 years, a growing number of studies have addressed the issue of the visual attention of children and adolescents with ASD to non-human animal targets. The primary aim of this scoping review was to conduct a state-of-the-art of the current literature concerning visual attention to animals in children and adolescents with ASD in order to further our understanding of the current knowledge concerning the specificities of interspecific interactions among youths with ASD. This scoping review led to the selection of 21 scientific publications from peer-reviewed journals. Each publication was examined with four objectives: (1) to review the methodologies and tools used to explore such questions; (2) to try to organize and summarize the main results; (3) to explore which factors may contribute to the differences in the observations reported in the studies; and, lastly, (4) to identify the main limits of the studies and the elements that future studies should take into consideration in order to better identify the specificities of visual attention towards animals versus humans in children and adolescents with ASD. Based on this scoping review, we highlighted that the methodologies used by the various authors differed, with all methods having their pros and cons, yielding information concerning the visual attention to animals displayed by children and adolescents with ASD. We discuss the implications of these visual attention specificities on the benefits of human-animal interactions in light of the literature concerning AAI, service dogs, and pets at home, especially in relation to the variation of several factors (e.g., age). The limitations of the selected studies in this scoping review are discussed. Lastly, we discuss the special status that an animal could endorse for youths with ASD compared to humans, both how they visually extract information from it and how they interact visually with it.

### 4.1. Methodological Variations: Implications and Contribution to Our Current Knowledge

The methodologies used to study visual attention varied greatly between the selected publications. The traits that varied the most frequently were the characteristics of the animal stimuli (i.e., picture or film formats, color or black and white). While methods were similar when the same experimental paradigm was used (e.g., eye-tracking tasks, fa ew seconds of exposure); the durations of exposure to the stimuli varied significantly when methodologies varied (from a few seconds for computerized paradigms to several tens of minutes in real life interactions). Three types of mediums for animal presentation were found, ranging from the simplest to the most complex: pictures of animals, videos, or real-life animals. The choice of stimulus format was related to the research question and the feasibility of the research. However, the type of medium for presentation may affect participants’ visual attention, as shown in an eye-tracking study on human faces. Indeed, when comparing results for static pictures to those for dynamic video clips, Speer et al. [111] found differences only for the dynamic condition: adolescents with ASD spent less time looking at people’s eyes than the TD group when the stimulus was dynamic, but groups did not differ because it was static. Saitovitch et al. [64] showed that general gaze abnormalities of children with ASD were detected better when dynamic stimuli were used (i.e., comparison of the same scene between film and picture).

Two main types of experimental contexts emerged during this review: so-called laboratory conditions (i.e., a generally unknown place for subjects in a standardized room) or naturalistic contexts (i.e., a familiar place for the child or adolescent in a less standardized environment). It is known that ASD frequently involves challenges in adapting to new environments, a need for immutability, and a dislike for changes (DSM-V, [1,112]). Some authors even go as far as to say that the nature of a situation can induce behavioral variations. For example, variations in the level of social stimulation can affect children with ASD’s joint attention [113]. These potential effects of both environmental changes and environmental stimuli on individuals with ASD’s responses and behaviors must be taken into account when interpreting and extrapolating results. In addition, replication of experiments with variations in experimental settings would be of interest to validate the generalizability of the results.

Three types of tools emerged from the selected publications as being the most frequently used for measuring visual attention: eye-tracking, “screen task” (i.e., experiment with a computer or a tablet), and behavioral coding of direct observations; the latter will be developed further below. Each of these three measurement methods makes it possible to explore different and specific aspects of visual attention. The first two tools (eye-tracking and screen tasks) involve attentional testing tasks that are regularly used in psychology to measure and compare attentional abilities. 

First, screen tasks can involve various experimental paradigms, such as visual search, detection of change, and preference selection. Some publications showed that, in visual search tasks, individuals with ASD performed better than matched controls, even for very difficult multiple conjunction searches [3,114,115]. However, children with ASD’s difficulties to track moving objects accurately or to visually process movements have been demonstrated [116,117]. In particular, youths with ASD have issues perceiving human actions and movements, as well as movements of non-living stimuli, when they are presented rapidly [118]. Studies using eye-tracking with social stimuli also showed that human faces were less attention-grabbing for people with ASD and that, when exploring a social scene, they spent less time looking at them while paying more attention to other elements of the visual scene (e.g., objects, bodies) [30,43,119]. Concerning human faces, people with ASD explored less socially relevant features of faces, and in particular, explored less the eye area than neurotypical people [81,111,120,121,122], but explored the mouth area more [38,123,124,125]. A study on neurotypical young adults reported that regardless of the species scanned (i.e., human, monkey, cat, or dog), the eyes were stared at the most frequently and inspected first, followed by the nose and mouth [126]. The eye area is important and informative for the recognition of facial expressions (i.e., a faster response to fear, surprise, and disgust in neurotypical individuals) [127]. Indeed, depending on the emotion displayed, the most informative area of the face changes. For example, expressing anger or sadness engaged more changes of the upper part of the face, while joy engaged more changes of the lower part of the face [128]. Strategies for exploring faces and focusing on relevant elements influence the processing of facial information [129]. Face exploration specificities of people with ASD could explain their processing atypicities, such as their difficulties to recognize facial expressions and, consequently, differences in social interactions [121,127].

Second, after the screen task, comes the eye tracking method. Youths with ASD found animals more attractive than humans, as measured in studies using either eye tracking in a natural context (dogs, [82]) or pictures (several species, [80,84]). Children with ASD focused more on the eye area of animal faces than on other facial areas [80,84,95] and looked more at relevant facial features of animal faces than of human faces (i.e., exploration with triangulation). One possible explanation for these differences between the exploration of human and animal faces by individuals with ASD could be related to the unique morphology of human eyes. Indeed, human eyes are characterized by a widely exposed and devoid of pigmentation sclera (i.e., the white part of the eye surrounding the colored iris), resulting in high contrast that facilitates identification of gaze orientation [130,131]. However, this characteristic is not found in either other primate species [130] or domestic species (e.g., dog, cat, guinea pig, rabbit, horse) that were used by the authors of the publications reviewed here. Animals’ eyes are generally more pigmented around the full iris, and the pupil is either vertical (cat), horizontal (horse, goat), or round (dog). We hypothesized that these differences between human and animal eyes could contribute to these differences in the visual attraction and exploration of the eye area for people with ASD.

Third, and last, a less expected method and original approach is the ethological approach. However, although it is pertinent, this last method has rarely been used to characterize interactions between humans, with and without ASD, and animals (e.g., [82,91,94,105]). For example, using an ethological approach in natural contexts allowed authors to reveal that animals were more attractive than humans for youths with ASD (dogs [82]). Using ethological methods in natural settings could, however, have the advantage of providing more ecologically valid and more complex results while avoiding the issues that lab experimentation may involve for children with ASD. Indeed, some authors, such as Klin et al. [30] and New et al. [89], recommended that more studies and experimental paradigms closer to everyday life conditions should be developed in order to better understand the whole autism spectrum. Studying real life social interactions observed in a naturalistic context is not only more representative but also allows a better description of different gaze features that are indeed present during interactions [132]. Additionally, combining eye tracking and ethological methods would enable further investigation of the link between cognitive attentional processes and interaction behaviors in order to provide a more global perspective on the mechanisms involved in human-animal interactions. This was recently developed by Dollion et al. [82] by using an on-board eye-tracking glasses system during a semi-standardized encounter while performing behavioral coding through direct observations.

Variability in methodology also concerned the species involved. The choice of species for the experimental paradigms focused mainly on dogs. Dogs are the most common pets within family households and are therefore more likely to be known by the participants (33% of the world’s pet-owning population have a dog [133]). The massive use of this species can be explained by its highly interactive characteristics and the fact that it is easily transportable and educable for audiences with less predictable behaviors and specific needs. Dogs also have good cognitive abilities, and in particular a heightened attention to human signals (e.g., understanding of pointing and human gaze direction), have a visual behavior similar to that of humans, and recognize human facial expressions [134,135,136,137,138]). Moreover, complex visual attention behaviors are involved in human-dog interactions, such as mutual gazes (e.g., [102,139]. These mutual gazes between a human and a dog were also observed between children with ASD and their pet dog [102], confirming the greater attention to dog faces shown by other studies of children with ASD [51]. A study by Davidson et al. [58] on children with and without ASD showed that the emotion recognition performances of children with ASD on dog faces were similar to those of children without ASD. Analyses of within-group results indicated that children with ASD recognized emotions more accurately on canine faces than on human faces; in contrast, no differences in accuracy were observed in the group of children without ASD. We argue that the processing of visual information from dogs does not seem to be impaired by ASD. Other species have been tested in addition to dogs to study children with ASD’s visual attention to animals. A wide variety of morphologies (i.e., size, color, breed) have been involved, as well as a wide variety of species: dogs, horses, cats, guinea pigs, monkeys, common elands, pigeons, cows, bears, camels, anseriforms, butterflies, and fish. However, only a few publications reported the characteristics of the animals used (e.g., age, breed, sex, type of facial expression, physical characteristics). In view of this diversity, it is important to consider the choice of species used in the experimental paradigms. One of the most important aspects of communication to establish between social animals is the willingness or ability to pay attention to one another [140]. Attention to others particularly affects the information extracted from them, and extraction variations (quality and quantity of information) vary from one species to the next, especially in inter-specific interactions (when the interacting individuals belong to different species). Indeed, when pet dogs and pet cats looked at children (with or without ASD) at home, they did not display the same type of visual attention to humans: pet dogs used more gazes and pet cats used more glances [102]. Conversely, ASD children did not display the same type of attention to them; they preferred to glance at pet cats. Furthermore, a recent study by Miralles et al. [56] showed that the empathic abilities of adults with ASD varied according to the species. Parents of children with ASD reported via a survey that dogs were the most cited species as a source of fear for children with ASD (9.2%), while only 0.4% of parents reported that their child was afraid of cats [141]. Dogs remain an attractive species for children with ASD who do not have a dog, as the majority of parents responded that their child liked dogs [73,77]. Other parents reported that their child with ASD was afraid of dogs but tolerated other pets better, such as cats or rabbits [73,77]. However, interestingly, the most common animal in families with children with ASD is a cat [142]. Only a few scientific studies have focused on identifying species-specific characteristics that are visually appealing to children. However, one study varying the degree of infantility of faces reported that neurotypical children preferred more infantile cats, but no difference was found for dog faces [143]. To conclude, all these aspects (e.g., a child’s attraction to a species, preferences for certain characteristics, visual contact with the species) can affect the interactions a child or adolescent with ASD can establish with an animal, as well as the results of the studies, and are therefore crucial points to consider in future studies.

When focusing on the influence of body orientation on human or animal stimuli, we need to pay attention to the orientation of the eyes. Very few studies have focused on manipulating this parameter and characterizing its impact on visual attention. Humans naturally prefer to look at someone when they know that they cannot be seen by them [144]. Authors have shown that children with ASD avoid the direct gaze of other humans and present exaggerated stress reactions to such gazes [145]. Valiyamattam et al. [84] found preferential attention to animal but not human faces with a direct gaze. As the aversive response to human eyes is not found for animal faces, the authors proposed that animal faces could be a source of greater social reward than human faces. This preferential orientation for animal stimuli had previously been reported in other experimental studies with various species (e.g., dog [75]; dog, cat, horse, cow, turtle, squirrel, parrot, rabbit, mouse, hamster [74]) and confirmed by neuroimaging data indicating greater activation of adolescents with ASD’s neural reward systems in response to animal stimuli (i.e., dog and cat [146]).

### 4.2. Implications of Visual Attention on the Benefits of Human-Animal Interactions

Attraction toward animals, reflected in heightened visual attention to the animal, is often used as a prerequisite for the attribution of a service dog to a family with a child or adolescent with ASD. Studies reported that integration of a service dog has numerous benefits for them: improved psycho-social development (e.g., improved social skills, psychological well-being), improved emotional wellbeing (e.g., reduction of the feeling of loneliness, bringing calm and comfort to stressful situations), and reduced problematic behaviors (e.g., [147,148,149,150,151,152]. Extended to the presence of pets at home, benefits have also been reported, such as improved emotional skills [77,142] and improved prosocial skills [91,153]. Even at school, integrating guinea pigs in a classroom resulted in emotional improvement, reduced stress, and improved social interactions with peers [154]. Nevertheless, the literature suggests that effects may be modulated by the children’s age when a pet arrives [91]. An animal could also be positive for youths with ASD in more sporadic contexts, such as animal-assisted interventions (AAI). Beneficiaries of AAI, for example, increase their positive social behaviors, present a more positive mood, decrease their problematic behaviors (e.g., stereotypies), and reduce their feelings of loneliness (e.g., [155,156]). In sum, increasingly, studies evidence the benefits of animals for youths with ASD, whatever the type of their exposure to them.

The specificity of the visual attention of youths with ASD to animals could contribute to these benefits. Indeed, when in contact with animals, people with ASD could develop, exercise, and reinforce visual strategies to gather social information that can favor subsequent social behaviors, for example, spontaneous gaze orientations at faces. Some authors suggest that the benefits of animals for individuals with ASD could result from a generalization phenomenon (e.g., [105]). In other words, repeated interactions with an animal would train, broaden, and enrich their behavioral repertoire and social skills, which they could then transfer to future interactions with other species, including humans [91,102,157]. Additionally, interactions with an animal involve behavioral regulation and are a good motivation to interact and learn. As shown by this review, children and adolescents with ASD are motivated to interact with animals and to pay heightened attention to them; both of these actions are key elements for learning [9]. To illustrate this hypothesis, a recent publication based on eye tracking exploration of human faces by Dollion et al. [85] showed that children with ASD living with a service dog directed less attention to face areas that were not relevant to facial expression processing, while they displayed more contrasted scanning strategies of relevant facial features according to the emotion displayed (i.e., joy versus anger).

Visual processing involves specific brain processing. For example, 5-year-old neurotypical children displayed distinct brain activations depending on the movement observed: either horse running (i.e., animal), human movement, or a virtual cartoon scene (i.e., virtual human) [158]. Whereas the centro-parietal areas of the left hemisphere were activated both during observation of real and virtual human movements, this was not the case when watching animal movements. This suggests that at least part of the cortical network may be specific to processing human movement (real and virtual). Moreover, studies using stimuli consisting of point light, allowing the perception of movement without the confusion of form, revealed that children with ASD did not show a preference for biological movements over non-biological movements [116,159]. Lastly, by examining ASD people’s brain activation while they watched pictures of faces, Whyte et al. [146] found a hypo-activation of the face processing network for human faces but not for animal faces. Therefore, the brain networks and processes engaged in the perception of human or animal stimuli do not seem to be the same. Consequently, we may hypothesized that ASD could specifically alter brain networks involved in the processing of human stimuli information.

Hormonal processes are also involved in social behaviors; for example, oxytocin, a neuropeptide secreted at the hypothalamic level, is notably involved in social affiliative and maternal attachment behaviors [160]. The secretion of this hormone in individuals with ASD presents abnormalities; their levels of oxytocin are lower than those of NT people [161]. When oxytocin levels were artificially increased (e.g., via intra-nasal administration), the performance of people with ASD’s social skills improved, e.g., higher scores for voice and face emotion meaning tasks [162,163,164], improved empathy skills [165], increased social attention, and social reactivity [166,167,168]. In addition, human-dog interactions lead to an increase in circulating oxytocin levels [169,170]. We can therefore hypothesize that interactions with a dog or a species valued by the individual could lead to the release of oxytocin, which would promote the expression and integration of social skills in people with ASD. 

### 4.3. Variation Factors

There is a large inter-individual variability in the attraction and interest that people—with or without ASD—have in animals, which is multifactorial in origin [171]. This variability in interest/attraction is also reflected in terms of visual exploration, as shown by different studies demonstrating a preference for animals with particular characteristics (i.e., more infantile facial features [143,172], which appear very early during development (as early as 3 years old). Although this issue of animal attractiveness may have direct consequences for interactions with animals and visual attention, it appears that this was not taken into consideration or evaluated in the studies included in this scoping review.

This scoping review highlights a clear lack of global information. First of all, the severity of ASD was poorly reported. Considering that seems important, one could argue that it could modulate the results, notably because the severity of ASD could be linked to visual alterations (e.g., having visual deficiencies such as blindness is linked to more severe autism phenotypes) [173]. Moreover, when ASD severity was mentioned, it appeared that participants were not representative of the whole autism spectrum, i.e., participants involved had mainly mild to moderate ASD severity. However, in real life, exposure to animals, either as pets, service dogs, or throughout AAI, is not limited to this sub-sample of ASD people, and the benefits could concern people on the whole autism spectrum. Other factors that could affect visual attention are the ages and emotions displayed by the animal stimuli. For example, the difficulties of children with ASD in identifying the age of non-human faces (primates, dogs, and cats) could vary more or less according to the age range used [38,59]. This same study suggested another factor of importance: familiarity with the stimuli. Indeed, repeated presentations of the same animal faces, when occurring, could certainly favor emotion identification on these faces by children with ASD. 

### 4.4. Limits

This scoping review allowed us to highlight three main limitations mentioned by the authors: sample size, lack of information concerning the participants, and limitations directly related to the protocol used. Other limitations were specific to only one or two studies, for example, related to the characteristics of the participants such as age, ASD severity (partial coverage of the spectrum), unbalanced sex ratio, or lack of a control group. The lack of information on comorbidities is particularly relevant and must be considered given their high prevalence in ASD. Indeed, approximately 70% of the individuals with ASD have one comorbid mental disorder, and 40% have two or more comorbidities (DSM 5, [1,174]).

Moreover, important elements to be considered when studying visual attention have been poorly investigated in these studies. The visual acuity of the participants was unfortunately never mentioned as a limitation, although a third of the publications took it into account in their inclusion criteria. Despite difficulties in assessing neurovisual disorders, it seems absolutely necessary to control this parameter before investigating visual attention skills. Indeed, the presence of visual acuity issues or neurovisual disorders could have major impacts on the results. In the literature on ASD, some authors used, for example, visual field tests and binocular visual pursuit [175]. Second, future research should be more precise concerning participants’ medication intake. Indeed, some categories of medication often prescribed in cases of comorbidity associated with ASD (e.g., ADHD), such as neuroleptics, benzodiazepines, and other types of antidepressants, can have adverse effects on vision (visual impairment, medicine referral site [176]. Additionally, future research needs to include a larger range of participants’ ages, as, at the moment, authors have focused mainly on 6- to 12-year-old children. Lastly, another piece of important information that was poorly reported concerned the presence of animals in the participants’ household as well as in their daily surroundings (e.g., “whether or not they own a pet”). Indeed, even when a child’s family does not own any animals, it may have the opportunity to be in contact with them through close relatives, as pets belong to our everyday environment (e.g., in France, slightly more than 50% of the households own at least one pet; [177]). In addition to owning a pet, regular participation in activities with animals (e.g., horseback riding, animal-assisted intervention) is common for individuals with ASD. All these elements could contribute to a familiarity bias when studying visual attention to animals. For example, the recognition of a dog’s emotions is modulated according to the cultural environment and, therefore, frequency of exposure to animals [178]. Moreover, Grandgeorge et al. [91] showed that children with ASD were more confident with an unknown guinea pig when they had pets at home. 

This scoping review highlighted the variation of the animal presentation format across studies, including both dynamic and static formats (mostly photos or real-life encounters and one film study). As all publications used different approaches to study visual attention, the results are not entirely comparable. Indeed, the results are study-specific, and there is a lack of replication of the methods used. Additionally, we must mention the large variability in the cuttings of AOIs (i.e., size, shape) and in the number of AOIs for studies using eye-tracking from one study to another.

A previous review of AAI addressed the lack of appropriate controls as the main limitation [155]. Here, the majority of studies had a control group, most often TD participants. The control group in a few studies was another group of children with ASD or children with other disorders (i.e., ADHD). However, most of the control groups had the same sex ratio or the same number of children as the test group. Nevertheless, even when chronological ages were close to those of the group of children with ASD, developmental age was never estimated in the studies included in our scoping review. 

## 5. Conclusions

For several years, Temple Grandin, a scientist with ASD, has spoken about her ease in understanding animals’ non-verbal communication cues compared to human cues [179]. This scoping review of the scientific literature on the visual attention of youths with ASD to animals highlighted the fact that, while interest in this topic is growing, it currently remains fairly new and poorly explored. It appears that animals have a special status for youths with ASD, both when visually extracting information from them and in interactions with them. The studies selected for this scoping review were mainly computer-based tasks or real-life encounters with an animal. The most studied component of visual attention was the visual exploration of animal stimuli. The second most studied topic was visual behaviors (e.g., gazes, glances), which were often integrated into behavioral profiles used to characterize interactions with an animal. Few studies focused on the early levels of visual attention, i.e., attentional capture. Altogether, these studies seemed to confirm that the visual attention of individuals with ASD to animals differs from their visual attention to humans. However, our scoping review also revealed a strong heterogeneity in the methods and animal stimuli used, as well as a lack of information regarding numerous relevant elements. Further research is needed to improve our understanding of the characteristics that determine the visual attention of children and adolescents with ASD to animals, including aspects of the integration of visual information about them. Further investigation of how individuals with ASD look at and integrate visual information from animals would improve our understanding of the real specificities in communicative and interaction skills associated with ASD.

## Figures and Tables

**Table 1:** Summary of the selected publications.

Authors	Year	Publication Title	Article Title
New et al. [89]	2010	*Neuropsychologia*	The scope of social attention deficits in autism: prioritized orienting to people and animals in static natural scenes
McPartland et al. [90]	2011	*Journal of Autism and Developmental Disorders*	Patterns of visual attention to faces and objects in autism spectrum disorder
Grandgeorge et al. [91]	2012	*Interaction Studies*	Children with autism encounter an unfamiliar pet: application of the strange animal situation test
O’Haire et al. [92]	2013	*PLoS ONE*	Social behaviours increase in children with autism in the presence of animals compared to toys
Guillon et al. [93]	2014	*Neuroreport*	Both dog and human faces are explored abnormally by young children with autism spectrum disorders
Grandgeorge et al. [94]	2015	*European Child and Adolescent Psychiartry*	Interest towards human animals and objects of children with autism spectrum disorders: an ethological approach at home
Muszkat et al. [95]	2015	*Frontiers in Psychiatry*	Face scanning in autism spectrum disorder and attention deficit/hyperactivity disorder: human versus dog face scanning
Grandgeorge et al. [80]	2016	*Human-Animal Interaction Bulletin*	Face processing of animal and human static stimuli by children with autism spectrum disorder: a pilot study
Grandgeorge et al. [96]	2017	*Scientific Reports*	Social rivalry triggers visual attention in children with autism spectrum disorders
Doherty et al. [97]	2018	*Developmental Science*	Visual search and autism symptoms: what young children search for and co-occurring ADHD matter
Strathearn et al. [98]	2018	*Development and Psychopathology*	Visual systemizing preference in children with autism: A randomized controlled trial of intranasal oxytocin
Germone et al. [99]	2019	*Autism*	Animal-assisted activity improves social behaviours in psychiatrically hospitalized youths with autism
Uccheddu et al. [83]	2019	*Animals*	The impacts of a reading-to-dog programme on attending and reading of nine children with autism spectrum disorders
Gale et al. [100]	2019	*Scientific Reports*	Children with autism show atypical preference for non-social stimuli
Yamashiro et al. [101]	2019	*Autism Research*	Shifting preferences for primate faces of neurotypical infants and infants later diagnosed with ASD
Grandgeorge et al. [102]	2020	*Frontiers in Psychology*	Visual attention patterns differ in dog vs. cat interactions with children with typical development or autism spectrum disorders
Avila-Alvarez et al. [103]	2020	*Health and Social Care in the Community*	Improving social participation of children with autism spectrum disorder: pilot testing of an early animal-assisted intervention in Spain
Valiyamattam et al. [84]	2020	*Frontiers in Psychology*	Do animals engage greater social attention in autism? An eye tracking analysis
Dollion et al. [82]	2021	*Journal of Autism and Developmental Disorders*	Visual exploration and observation of real-life interactions between children with ASD and service dogs
Scheerer et al. [104]	2021	*PloS ONE*	Attention capture by trains and faces in children with and without autism spectrum disorder
Dollion et al. [105]	2022	*Anthrozoos*	Characterization of children with autism spectrum disorder’s interactions with a service dog during their first encounter

**Table 2:** Summary of the participants’ characteristics.

Authors	N ASD	Diagnoses	Diagnostic Tools	Additional Tools for ASD Measurement	Age of ASD Subjects (Mean ± SD)/(Range)	Gender of ASD Subjects	Pet Ownership
New et al. [89]	31	ASD	ADI-R, ADOS	VABS	10.8 ± 3.4 yo	30 M; 1 F	NA
McPartland et al. [90]	15	ASD	ADI-R ADOS clinical diagnosis DSM-IV-TR criteria for AS	The VABS—Survey Form based on parent reports, the Social Competence Questionnaire, and SRS	14.5 ± 1.7 yo (12.0–16.6)	13 M; 2 F	NA
Grandgeorge et al. [91]	27	ASD	ADI-R, DSM-IV criteria	CARS, Vineland for a part of them.	9.6 ± 1.8 yo (6–12)	27 M	19 with pets
O’Haire et al. [92]	33	ASD (n = 7) AS (n = 14) PDD-NOS (n = 5) and AD (n = 7)	All had a previous diagnosis established by experts	SCQ and SSRS for a part of them completed by parent and teacher	9.4 ± 2.3 yo (5.2–12.1)	24 M; 9 F	All ASD children
Guillon et al. [93]	19	ASD	ADOS-G and ADI-R performed by at least one clinical psychologist	Mullen Scales of Early Learning	39.6 ± 10.5 months (24–60)	15 M; 4 F	NA
Grandgeorge et al. [94]	31	ASD	The ADI-R by independent psychiatrists	VABS, CARS, and ICD-10 criteria	9.5 ± 1.8 yo (6–12)	30 M; 1 F	22 with pets
Muszkat et al. [95]	15	ASD	CARS and DSM-V criteria	NA	11.6 ± 2.7 yo	13 M; 2 F	NA
Grandgeorge et al. [80]	12	AD (n = 3) AS (n = 9)	ADI-R, ADOS	NA	136.6 ± 14.7 months (87–187)	10 M; 2 F	Some with pets, but number of concerned participants not specified
Grandgeorge et al. [96]	S1: 20 S2: 9	ASD	S1 and S2: A parent-reported diagnosis of ASD and a medical report of ASD using ADI-R1, and DSM-IV criteria	SCQ completed by parents	S1: 7.6 ± 1.6 yo S2: 13.7 ± 2.3 yo	S1: 9 M; 1 F S2: 8 M; 1 F	NA
Doherty et al. [97]	98 HR	56 HR Typical 28 HR Atypical 14 HR ASD	ADOS-2, ADI-R, and DSM-5 criteria.	Vineland-II, Mullen Scales of Early Learning, SBRI	38.8 ± 1.57 months	55 M; 43 F	NA
Strathearn et al. [98]	16	ASD	ADI-R, ADOS, and DSM-IV criteria	VABS	12.8 ± 3.4 yo (8.2–19.0)	16 M	NA
Germone et al. [99]	67	ASD	ADOS-2 criteria	SCQ	11.7 ± 3.5 yo	53 M; 13 F; 1 NA	23 with dogs, 15 with cats, 1 with rabbits, 1 with guinea pigs, 15 with multiple types of pets
Uccheddu et al. [83]	5	ASD	DSM-V criteria and tools required by the Ministry of Health	NA	7.60 ± 2.3 yo (SE) (6–11)	4 M; 1 F	NA
Gale et al. [100]	S2: 19	ASD	ICD-10 criteria	CARS-2, VABS	S2: 58.2 ± 21.1 months (26–96)	S2: 15 M; 4 F	None of the participants had pets
Yamashiro et al. [101]	S1: 15 ASD (according to the age of the children tested, N varies between 7 and 11). S2: 7 ASD (according to the age of the children tested, N varies between 5 and 7)	ASD	Toddler Module of ADOS	NA	S1 and S2: NA (6–18 months), test at 6 (only S1), 9, 12 months	NA	NA
Grandgeorge et al. [102]	22	ASD	ADI-R, DSM-IV criteria	ICD-10 criteria	10.1 ± 2.1 yo (6–12) with a dog and 7.5 ± 2.2 yo (6–12) with a cat	14 M	14 with dogs, 8 with cats
Avila-Alvarez et al. [103]	19	ASD (n = 15), probable ASD (n = 4)	DSM V criteria	NA	46.2 ±12.6 months (30–66)	13 M; 6 F	7 with dogs and 3 with cats
Valiyamattam et al. [84]	21	ASD	Parent and/or teacher reported diagnosis of ASD	SCQ, SRS-2	10.3 ± 1.60 yo (5–12)	16 M; 5 F	NA
Dollion et al. [82]	S1: 16 S2: 6	ASD	Parent reported diagnosis of ASD	CARS	S1: 8.5 ± 0.7 yo S2: 9.3 ± 1.1 yo (6–15)	S1: 14 M; 2 F S2: 3 M; 3 F	NA
Scheerer et al. [104]	S1: 29 S2: 10	ASD	British Columbia (BC) clinical diagnostic report ADOS, ADI-R	AQ parent-report questionnaire	S1: 10.35 ± 1.93 yo (6–14) S2: 9.02 ± 2 yo (6–12)	S1: 20 M; 9 F S2: 8 M; 2 F	NA
Dollion et al. [105]	20	ASD	Parent reported diagnosis of ASD	NA	8.6 ± 27 yo (3–12)	16 M; 4 F	NA

Legend: S: study; SE: Standard error; ASD: autism spectrum disorder; PDD-NOS: Pervasive Developmental Disorder-Not Otherwise Specified; AD: Autism Disorder; AS: Asperger Syndrome; HR: high risk; SBRI: Stereotyped Behaviors and Restricted Interests; SSRS: Social Skills Rating System; SRS: Social Responsiveness Scale; AQ: Autism quotient.

**Table 4:** Summary of stimuli characteristics, results and limits mentioned.

Stimuli Characteristics				Results	
Authors	Species/Breed of Animals (n = )	Type of Material (e.g., Screen Tablet, Real Interaction)	Animal Known or Unknown to the Subject	Main Significant Results about Visual Attention	Main Limits Mentioned
New et al. [89]	Color photographs of natural scenes; 14 scenes for the animal semantic category. Target animals were primates of the Catarrhini sub-order (n = 1); common Eland (n = 1), and pigeon (n = 1)	Computer screen SS: NA SD: NA	Unknown	Children with ASD exhibited robust social attentional biases for categorical animacy—detecting changes faster and more reliably in people and animals compared to artifacts and plants.	None were mentioned.
McPartland et al. [90]	Digitized standardized grayscale images: Monkey faces (n = 10) presented bilaterally and symmetrically	Computer screen SS: 21-inch SD: 15° × 19° covert	Unknown	Both groups devoted greater attention to the upper versus lower AOI for both human and monkey faces. No other effects or between-group differences were detected.	Study design limits inferential power and generalizability. Influence of the particular visual characteristics of a homogenous stimulus class on viewing patterns could not be explored. Wide range of participants’ age, developmental effects could create a bias. Measures of fixation were limited to theoretically-defined AOIs. Aspects of the experimental design may influence the results. Extended viewing times of 8 s may have affected viewing patterns.
Grandgeorge et al. [91]	Guinea pigs (n = 4)	Real interactions	Unknown	First gaze of the children with ASD upon entering the room with the guinea pig was mostly towards the animal with 66.7% of the children with ASD having looked at the animal.	None were mentioned.
O’Haire et al. [92]	Guinea pigs (n = 2 per observation; n = 30 in total)	Real interactions	Unknown at the beginning (8 weeks of experiment, twice-weekly session)	Children with ASD looked at the toys significantly more often than at the animals.	Lack of information about participants’ cognitive functioning or IQ. Difference in the availability of animals (n = 2) vs. toys (large variety).
Guillon et al. [93]	Grayscale photographs: dog faces (n = 12) with negatively (n = 4), neutrally (n = 4), and positively (n = 4) valenced expressions	Computer screen SS: 17-inch SD: 19° × 20° covered	Unknown	There was no gaze bias to the left visual hemifield for the ASD group (whether human or canine), while the TD group manifested a left bias for both species. Emotional valence had no effect on the laterality index of the first fixation for both human and canine faces.	Children’s manual and ocular dominances were not evaluated.
Grandgeorge et al. [94]	Guinea pig (n = 4)	Real interactions	Unknown	TD children performed more visual behaviors towards the animal compared to ASD children (eye direction toward the animal: 48.8 ± 17.2% vs. 79.7 ± 9.6%, respectively). Children with ASD looked more at the animal than they touched it. The visual index of focusing only on the pet was higher for TD children than for ASD children.	Limitation due to cross-sectional design.
Muszkat et al. [95]	Color photographs: dog faces with neutral expressions (n = NA)	Computer screen SS: 23-inch SD: NA	Unknown	All children looked more at dog pictures than at human ones. The eye area was looked at the longest compared to the mouth area in all children. The duration of fixation on the eyes was lower in the ASD group than in the TD group. When children with ASD looked at human and dog faces, they did not manifest the left visual hemifield preference observed in the TD group.	Small sample size, as well as the demographic and clinical heterogeneity of the samples.
Grandgeorge et al. [80]	Black-and-white pictures of dog (n = 2), cat (n = 2), and horse (n = 2) faces in a natural context	Computer screen SS: NA SD: resolution of 500 × 800 pixels	Unknown	Children with ASD spent less time looking at the eyes on all animal pictures than TD children. No difference between the two groups in time spent looking at the mouth or ears. All children looked longer at humans’ mouths than at animals’ mouths. Children with ASD looked more at the eyes than at the mouth and ears of dog pictures, and they looked longer at mouths than at ears. Children with ASD looked more at the eyes than at the mouths and ears of cat pictures. They looked more at the eyes and mouth than at the ears of the horse pictures.	Small sample size of the ASD group. Pictures were not standardized in terms of AOI sizes and/or background. A small number of pictures used.
Grandgeorge et al. [96]	S1: Service dogs (n = 9): three Labrador retrievers and six golden retrievers (eight males; mean age ± SD: 23.8 ± 0.5 months) S2: Service dogs (n = 2): one male Labrador retriever and one female golden retriever (mean age ± SD: 23.9 ± 0.2 months)	Real interactions	Unknown	S1: children with ASD alternate more glances between the service dog and the dog trainer when the child is out of the service dog-animal trainer interactions. Compared to the control group, children with ASD glanced more towards the dog-trainer dyad when they both had a close interaction without visually attending to the child. S2: the three repetitions of the procedure showed that children with ASD maintained strong visual attention towards the service dog-trainer dyad when the child was out of the interaction. Most instances of joint attention between children with ASD and the trainer were on the service dog (85%).	None was mentioned.
Doherty et al. [97]	Colored pictures of animals: bears, camels, cats, cows, and dogs (same picture for each species, n = NA)	Touchscreen monitor. SS: 14.94 × 11.94-inch SD: 3.18 cm × 2.39 cm	Unknown	In multi-target cancellation tasks with more complex targets/distractors, ASD symptoms were associated with more disorganized visual search across conditions and poorer search performance, for categorical search in particular. ASD symptom severity did not correlate with search performance, but it did correlate with poorer categorical search.	Diagnosis is not confirmed with a gold standard measure. The three experimental conditions were administered in a fixed order, allowing for potential order or fatigue bias. Presence of missing data for 10 children. Small sample size.
Strathearn et al. [98]	four images of four levels of picture systemizing (systematization is the level of organization of the image (e.g., a high level of systematization means that the elements in the scene are highly organized)) positioned in each quadrant of each slide. Systemizing Picture Task using real-life images of animals (not specified, but provide examples of images with anseriform birds (n = 4))	Computer screen SS: 17-inch SD: NA	Unknown	In the placebo condition, children with ASD showed a visual preference for more highly systemized images. There was a significant linear trend for an increase in the duration of fixation in children with ASD with increasing levels of picture systemizing, while no such effect was seen in the control group.	Systemizing Picture Task has not yet been validated. Physical aspects of the pictures, such as color intensity or background features, were not controlled for and may have influenced gaze preference. Lack of systematic information on psychiatric comorbidity.
Germone et al. [99]	Therapy dogs (n = 6; all females): golden retriever (n = 2), Border Collie/golden retriever Mix(n = 1), King Charles Spaniel Mix (n = 1) and Labrador Mix (n = 2)	Real interactions	Unknown at the beginning (NA total session, two sessions per week)	Children with ASD in the control condition looked more at the toy; children with ASD in the experimental condition looked more at the dog.	Participants were not randomly assigned to experimental (AAA with a dog) or control (toy) conditions. Intra-individual variability of participant’s behaviors during their hospital stay.
Uccheddu et al. [83]	Therapy dogs (n = 2): 2 females, mixed-breed dogs	Real interactions	Unknown at the beginning (10 weekly sessions for the experimental group)	Questions to parents concerning their child’s “attention to dogs” in their daily lives revealed no differences between children with ASD in the experimental condition (reading in the presence of a dog) or the control condition (reading without the dog).	Small sample size. The effects of confounding variables (e.g., parenting styles, comorbid outcomes such as anxiety) were not controlled for. Subjectivity due to parental report. Potential desirability bias in the answers to the questionnaire.
Gale et al. [100]	Videos: dogs’ faces (n = 6)	Tablet screen SS: NA SD: 7 cm × 7 cm, and after touch, 13 × 13 cm.	Unknown	Children with ASD preferred non-social stimuli (geometric images) to dog faces (geometric images were indicated as preferred 69.9 ± 14.8% of the time compared to 52% for dog faces). Preference for non-social stimuli remained high for children with ASD, while it decreased for TD children across sessions.	Many of the same participants took part in the three studies. There was no cognitive assessment of participants. Human stimuli were only Caucasian adults, while the participants had various ethnicities.
Yamashiro et al. [101]	Grayscale photographs placed on a gray background: adult female rhesus monkey faces (n = 2)	Computer screen SS: 29 cm × 47 cm (equivalent 22-inch). SD: 16.5 × 20 cm (adjusted to screen size)	Unknown	6-month-old infants later diagnosed with ASD only preferred human faces over non-faces and did not prefer monkey faces over non-faces until reaching 9 months of age. Compared to TD infants, infants later diagnosed with ASD showed a greater downturn in their preference for primate faces compared to non-faces, as well as in their total looking time for both primate faces between 6 and 18 months.	None mentioned.
Grandgeorge et al. [102]	Dogs (n = 14) (six females) Cats (n = 8) (five females)	Real interactions and questionnaires	Known	Mutual gazes with cats were rare between children of both groups. In both groups, cats and children initiated glances and mutual gazes equally often. Children with ASD directed more gazes and glances towards their cats than their dogs. Parents of both groups reported that their children had fewer visual interactions with cats than with dogs.	The length of the videos varied due to the ecological situation.
Avila-Alvarez et al. [103]	Therapy dogs (n = 5; four males and one female): Labrador retrievers (n = 3), Galician Shepherd Dog (n = 1), Spanish Water Dog breed (n = 1)	Real interactions	Unknown at the beginning (total of 9 weekly sessions)	Significant increase in the frequency of children with ASD’s eye contacts with the dog and in their participation in activities with the dog across sessions.	Absence of a control group. Lack of information, such as the severity of ASD. Non-probability convenience sample (Sample of participants not representative of the whole population (i.e., non-random); here for example, the sample was recruited by a non-random technique and participants were predominantly male). Inclusion of children with probable ASD. Variability in the number and length of AAI sessions.
Valiyamattam et al. [84]	Color photographs of animals against a constant gray backdrop: dogs (n = 8), cats (n = 8), horses (n = 2) and cows (n = 2)	Computer screen SS: 21.5-inch SD: 29.5° × 32.5°	Unknown	Children with ASD showed significantly less visual attention to the face and eye area, left and right eyes, as well as to the mouth area on both human and animal pictures combined compared to TD children. Children with ASD showed greater visual attention to the face and eye region of animal pictures compared to human pictures. Children with ASD showed greater visual attention toward the mouth and screen areas on human pictures compared to animal pictures. Children with ASD showed greater visual attention to the eye regions and face of animals for front-facing pictures compared to averted-facing images. No significant difference was observed in TD and ASD children’s visual attention to the mouth area between front and averted-facing human and animal pictures.	ASD children’s mental age was lower than that of TD children. The number of males was higher in the children with ASD group.
Dollion et al. [82]	S1: Service dogs (n = 15; 10 females): Labradors (n = 5) Labernois (n = 3) and Saint-Pierre (n = 5) S2: Service dogs (n = 4; all females): Labradors (n = 2), Labernois (n = 1), Saint-Pierre (n = 1)	Real interactions	Unknown	Children with ASD spend more time looking at the service dog, particularly its head, than at other elements of the visual scene (mean percentage of fixation duration: 44.4 ± 3.2% on the service dog and 31.4 ± 2.5% on the service dog’s head). Some of the ASD children’s interaction behaviors correlate with their visual exploration (e.g., attention to animate/inanimate stimuli). Children with ASD who looked more at the social/animate targets showed more engagement in their interaction with the service dog.	Small sample size. Participant selection was partly based on their initial attraction towards dogs. Choice of AOIs for analysis (i.e., the decision applied to select and delimit AOIs may have had a limiting effect on the collected data).
Scheerer et al. [104]	S1 and S2, gray-scale pictures: butterfly (n = 1) and fish (n = 1)	Computer screen SS: 23-inch SD: 3 × 3 cm	S1 and S2: Unknown	When the target was absent, both groups of children were more accurate but slower at detecting the absence or presence of the butterfly target. For both groups, the butterfly target was fixated on the majority of targets present in the trials. Children with ASD detected the butterfly more slowly and took longer to fixate the butterfly than TD children.	Age of participants. Inter- and intra-individual variability with regard to past experience with the stimuli and interest in the stimuli. The task was too easy. Small sample of children for the eye-tracking part.
Dollion et al. [105]	Service dog (n = 18; 5 females): Labrador (n = 8), Labernois (n = 3), Saint-Pierre (n = 7)	Real interactions	Unknown	The service dog was the preferred target of ASD children’s gazes. Younger children with ASD gazed less at the service dog than older children. Two profiles of children with ASD were observed: one group interacted distally with the service dog and looked less at it compared to the second group, which interacted more proximally and looked more at it.	All participants were on the waiting list to receive a service dog (potential bias of attraction towards dogs). Narrow range of autism severity. Absence of information concerning the presence/absence of pets at home, previous experience with animals, and frequency of activities with animals. The first version of the CARS scale was used.

Legend: S = study, SS = screen size; SD = Stimuli Dimension, NA = Unknown.

## Data Availability

The data presented in this study are available in article.

## Appendix A

### Appendix A.1. Search Equation

The equations were composed from keywords and linked by the Booléen operators (AND) between each category and within each category linked by (OR) and the search was carried out on the title and/or the abstract: visual attention (visual capacity, visual capacities, visual attention, visual focus, face, facial, vision, attention, perception, gaze, gazing, glance, glancing, look, looking), type of population (child, children, toddler, kid, adolescent), disorder (ASD, ASD, Autism, autistic, PDD, Asperger’s) the visual target (animal, pet, horse, dog, cat, guinea pig, monkey, ape, bird) The equations have been adapted to the search engine’s capabilities. We excluded keywords that could be associated with ASD or attention but not wanted in the search with their plural (e.g., techno, virtual, electro digital, robot, vaccine, pesticide, urine, gene, pharma, pollution, acid). (((visual capacity[Title/Abstract] OR visual capacities[Title/Abstract] OR visual attention[Title/Abstract] OR visual focus[Title/Abstract] OR face[Title/Abstract] OR Facial [Title/Abstract] OR vision [Title/Abstract] OR attention[Title/Abstract] OR perception[Title/Abstract] OR gaze [Title/Abstract] OR gazing [Title/Abstract] OR glance[Title/Abstract] OR glances[Title/Abstract] OR glancing[Title/Abstract] OR look[Title/Abstract] OR looking[Title/Abstract] OR eye [Title/Abstract]) AND (Child[Title/Abstract] OR children[Title/Abstract] OR Toddler[Title/Abstract] OR Toddlers[Title/Abstract] OR Kid[Title/Abstract] OR Kids[Title/Abstract] OR adolescent[Title/Abstract] OR adolescents[Title/Abstract]) AND (ASD[Title/Abstract] OR TSA[Title/Abstract] OR Autism[Title/Abstract] OR Autistic[Title/Abstract] OR TED[Title/Abstract] OR Asperger[Title/Abstract)) AND (animal[Title/Abstract] OR animals[Title/Abstract] OR pet[Title/Abstract] OR pets[Title/Abstract] OR Horse[Title/Abstract] OR Horses[Title/Abstract] OR dog[Title/Abstract] OR dogs[Title/Abstract] OR cat[Title/Abstract] OR cats[Title/Abstract] OR guinea pig[Title/Abstract] OR monkey[Title/Abstract] OR monkeys[Title/Abstract] OR ape[Title/Abstract] OR apes[Title/Abstract] OR bird[Title/Abstract] OR birds[Title/Abstract])) NOT (techno*[Title/Abstract] OR virtual[Title/Abstract] OR electro*[Title/Abstract] OR digital[Title/Abstract] OR robot[Title/Abstract] OR robots[Title/Abstract] OR vaccine[Title/Abstract] OR vaccines[Title/Abstract] OR pesticide[Title/Abstract] OR pesticides[Title/Abstract] OR urine[Title/Abstract] OR gene*[Title/Abstract] OR Pharma*[Title/Abstract] OR pollution[Title/Abstract] OR acid[Title/Abstract])
